# The influence on resection line during supracervical hysterectomy: physiological extension of endometrial cells in the cervix uteri

**DOI:** 10.4274/jtgga.galenos.2021.2020.0209

**Published:** 2021-02-24

**Authors:** Carolin Spuentrup, Elke Wendt, Marc Banerjee, Jörg Schmitz, Martin Hellmich, Günter-Karl Noé

**Affiliations:** 1 Department for Gynecology, Obstetrics and Reproductive Medicine, University of Witten/Herdecke, Witten, Germany; 2 Department for Gynecology, Obstetrics and Reproductive Medicine, Saarland University Hospital, Homburg, Germany; 3 Department of Obstetrics and Gynecology, Rheinlandklinikum Dormagen, Dormagen, Germany; 4 Media Park Clinic, Cologne, Germany; 5 Institute of Pathology, Grevenbroich, Germany; 6 Institute for Medical Statistics and Epidemiology, University of Cologne, Germany

**Keywords:** Endometrial glands, cervical glands, uterine isthmus, supracervical hysterectomy, spotting

## Abstract

**Objective:**

A straight resection of corpus uteri using the sacrouterine ligament as landmark is a common method during supracervical hysterectomy. Subsequent spotting rates of up to 25% suggest the existence of residual endometrial glands in the remaining cervical tissue, casting doubt on the landmark qualities of the sacrouterine ligament. Fifty-one females who underwent total laparoscopic hysterectomy for benign diseases were investigated.

**Material and Methods:**

Macroscopic uterine parameters were determined during operation. First appearance of endometrium cells, complete disappearance of endometrial cells in the cervix and others were measured microscopically with reference to the external cervical orifice. Associations were described using odds ratio with 95% confidence interval and p-value <0.05.

**Results:**

The region of the cervix, in which exclusively cervical glands are found, is relatively small but varies considerably around the mean (mean, 23.3 mm, range, 10 to 35 mm). In this cohort in a remnant cervical stump of 23 mm length, endometrial glands would be found in 51%. There was no correlation between full cervical length and uterine parameters but smaller uteri tended to be associated with deeper endometrial penetration.

**Conclusion:**

There is a discrepancy between common definition and histological findings concerning the cervix uteri. Our findings indicate that the sacral uterine ligament is not suitable as an anatomic landmark for the laparoscopic supracervical hysterectomy operation. Regarding the distribution pattern of endometrial glands in the isthmic zone, a deep conical excision seems to better prevent subsequent spotting than a straight resection with thermocoagulation of the remaining cervical canal.

## Introduction

In northern Europe 41.5% of hysterectomies are performed as a supracervical hysterectomy (1). Patients often prefer this method as only the diseased part of the uterus is removed, for example the corpus uteri in the case of heavy bleeding, and the risk of intraoperative complications, such as lesion of the ureter or intraoperative blood loss, is reduced. In addition, when preserving the cervical tissues, the operation can be performed more easily and faster. A further benefit is that the remaining cervical structures may be useful for fixation in case of later prolapse.

**Figure 1:**
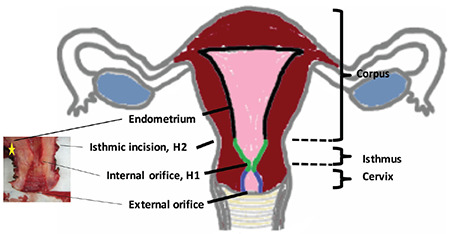
Anatomical subdivisions of uterus The common anatomical doctrine is demonstrated schematically. The small picture on the left shows the lower uterine segment of one of our specimens. The yellow star indicates the sacrouterine ligament, used as the resection line in the case of laparoscopic supracervical hysterectomy

One disadvantage of supracervical hysterectomy is the risk of persistent spotting. The uterine isthmus is considered to be the border between corpus and cervix (2,3). Surgeons often use the sacral uterine ligament as a macroscopic landmark of the uterine isthmus (Figure 1) (4,5). The excision of the corpus uteri is frequently performed as a straight resection preserving the ligament, followed by thermocoagulation of the remaining cervical channel (4,5). Endo-loops and slings simplify this procedure (6). To date, few data concerning the histological structure of the isthmus have been reported; however, the data available show that the isthmic region is more than just the border between cervix and corpus uteri (2,3,7,8,9). Endometrial cells are regularly found coexistent with cervical stroma in the uterine isthmus (2,3,10). Therefore, it is not unexpected that spotting rates of up to 25% have been reported after supracervical hysterectomy using a straight resection above the sacral uterine ligament (11). Forty percent of patients suffering from spotting after laparoscopic supracervical hysterectomy (LSH) report a reduced quality of life (12). Spotting can be reduced by a conical excision of the cervical canal, but is nevertheless reported to persist in 6% of cases (10,13).

It is likely that the anatomical correlates are remnant endometrial glands. These glandular tissues will respond to female hormones and cyclic bleeding may be induced. If this hypothesis is true, the risk of later endometrial carcinoma cannot be excluded but appears to be very low. Only a few cases of new endometrial carcinoma in the remnant stump have been reported to date (14,15).

The aim was to determine the extension of the isthmic region and to investigate the extent of the explicit cervical part, as defined by the presence of cervical glands and the complete absence of endometrial glands. Additionally, we attempted to find a correlation between uterine length or weight and the tissue limits of the presence of endometrial glands.

## Material and Methods

The uteri of 51 patients who underwent a complete hysterectomy were analysed in the Pathological Institute of Hagen Grevenbroich. Only patients with benign disease of the uterus were included in the study, including uterus myoma (n=13), adenomysosis (n=15), both myoma and adenomyosis (3) or other indications (n=20). Patients suffering from endometrial or cervical cancer were excluded.

Total uterus length and weight of the uterus were measured before further pathological investigations were undertaken. Intracaval length was reported, if measured intraoperatively (n=47).

The study was approved by the Institutional Ethical Committee University of Witten/Herdecke (approval number: 34/2011, date: 30.05.2011). Informed consent was obtained.

### Histopathological reprocessing

A longitudinal median cut through the uterus was performed. The area of interest area of the cervix, that was the area between the external orifice and the area above the isthmical incision, was identified on one half of the uterus. Matched, longitudinal sections were obtained from the correlating area of the other uterus half.

These sections were prepared for further analysis (Figure 2a). If no endometrial glands were found in this first slide, the slide above was also analyzed (Figure 2b). The configuration at the interrupted areas was not always possible over the whole diameter of the cervix due sectioning artefact. The best configured parts were used for histological estimation of cervical and endometrial glands in these cases.

As a reference point for measurement, the perpendicular to the external orifice of cervix was used. Measurements were performed using an optical scale.

The distance between the external orifice of the cervical canal and the solely endometrial glands with absence of cervical glands was measured microscopically (Figure 3a, Table 1). The distance between the external orifice of the cervical canal and the absence of endometrial glands was also determined (Figure 3a, Table 1). The length of the transition zone in which both type of glands (cervical and endometrial) were present, was derived from measurements (Table 1, Figure 3b).

### Statistical analysis

Associations (length, intracaval length, weight of uterus vs. extension of endometrial cells) were described using odds ratios and 95% confidence intervals. P-values less than 0.05 were accepted as statistically significant. Statistical evaluation was performed with SPSS 18.0.2 (SPSS Inc., Chicago, IL, USA).

## Results

The area of containing exclusively cervical glands was relatively small with a medium length of 23.3 mm (range, 10-35). In our study 17.6% of patients (9/51) had a cervix smaller than 20 mm (Figure 3a, b).

In this study the isthmic zone, characterized by the co-existence of both cervical and endometrial glands, had a medium depth of 2.8 mm (range, 0.5-8).

After supracervical hysterectomy the mean remaining cervical length was 2.3 cm. Measurement by ultrasound showed the cervical stump length to vary between 2.0 and 2.5 cm. Endometrial glands were present in 51% of cases (Figure 3c).

There was no correlation between the extension of endometrial glands and uterine length or weight. However; there was a tendency for small uteri to correlate with a deeper extension of endometrial glands into cervical tissues.

## Discussion

The extent of the isthmic area is variable but tends to be small. In some cases, the areas containing solely cervical structures measured only 10 mm. It can be supposed that if the remaining cervical part following LSH measured on average 2.3 cm, endometrial glands would be found in 51% of cases. No correlation with other factors that could be estimated before the operation, such as uterine weight or length, was found.

Only a few studies have investigated the histophysiology of the uterine isthmus. In 1953, Frankl (2) complained of the ignorance of the necessity of a threefold division of the uterus into corpus, isthmus and cervix. He defined the isthmus as the “hollow space of the cranial part of the cervix” with a different histological structure, similar neither to corpus nor to cervix. In 1958, Ober described his findings of unexpected endometrial material after strict cervical surgery and proposed an area in which the characteristic cells of corpus and cervix coexist (3). He detailed these findings and characterised the uterine isthmus as an independent region with smaller and fewer endometrial glands and more compact stroma. Additionally, he defined lines, marking the borders between cervical part and isthmic part (inner cervical orifice, H1) and the isthmic part and the endometrial part (isthmic incision, H2, Figure 1). This threefold division is now part of common anatomic doctrine, but there is still a lack of knowledge concerning the definition, the extension and borders and especially the macroscopic landmarks of the transition zone. Hoogduin et al. (16) tried to identify the borders using immunohistochemical markers. These authors reported that all three parts of the uterus can be separately identified by immunohistochemical markers, and that the transition from cervical cells to the isthmic part is histologically abrupt.

Few data concerning the risk of endometrial carcinoma following supracervical hysterectomy are available (14,15,17). This risk is generally considered to be unimportant. Most authors assume the implantation of endometrial cells during the removal process. Histologically, there is no consensus concerning the reaction of the isthmic endometrial glands to hormones (2,3,7). Our findings indicate that isthmic endometrial glands, at least in part, react to ovarian stimulation. It is very unlikely that endometrial carcinoma will occur after supracervical hysterectomy, but it cannot be completely ruled out.

In November 2014, the Food and Drug Administration (FDA) abandoned power morcellation for women having uterine fibrinoids due to the risk for spreading occult sarcoma. This rule was based on the finding that one of 458 operated patients suffered from occult leiomyosarcoma. A worldwide discussion concerning the sense of supracervical hysterectomy ensued and several surgeons returned to total hysterectomy. Meanwhile, several studies showed that the risk for dissemination of occult malignant tissue during morcellation was much lower (one of 1,550 patients, 0.064%) compared to the risk acted on by the FDA (18,19,20). Most national societies developed guidelines to reduce the risk for intraoperative morcellation of sarcoma by giving detailed preoperative diagnostic rules. These developments have led to the widespread re-adoption of supracervical hysterectomy.

There are pros and cons that should be taken into account when considering the procedure to be undertaken; supra-cervical hysterectomy versus total hysterectomy. After total hysterectomy no more spotting will appear and if a complete removal, without further morcellation is possible, the risk of dissemination of occult sarcoma is very low. However, preparation is more complex, associated with a higher risk of bleeding and complications or postoperative infection and insufficient suture. In case of later descensus surgery, there is likely to be only low-quality vaginal tissue as fixation point.

In contrast, supracervical hysterectomy is associated with a lower complication rate and lower operation time, but there is a small (0.064%) risk of spreading sarcoma. New systems allow morcellation in a bag, which reduces the remaining risk. Finally, it should be mentioned that several studies could show that occult sarcoma found after morcellation are not associated with a worser outcome (19,20).

The preservation of the cervical stump has a positive effect on patients physical and psychological well-being, as women prefer the integrity of vagina and pelvic floor. Additionally, it offers a stable potential fixation area in case of later descensus surgery. The possibility of postoperative spotting rate should be discussed preoperatively. Spotting rates after a straight resection and coagulation of the remaining cervical canal vary between 6-25%.  Banerjee et al. (21) analyzed spotting and infection rates over a six-month follow up after straight resection and coagulation versus conical excision and coagulation. The spotting rate after straight resection and bipolar coagulation was 21.6% (19/88) versus only 5.9% (5/85) after conical excision and coagulation. No difference concerning stump infection rate was found (straight resection 6.8% (6/88) vs 5.9% (5/85) for conical excision) (21).

**Figure 4:**
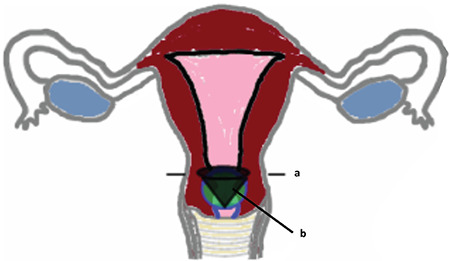
Schematic demonstration of conical excision Former resection line (a) vs a proposed new resection zone (b): A conical excision of the lower uterine segment with preservation of only the external 10 mm of cervix is more likely to result in a resection of the complete isthmus

Supracervical hysterectomy has not been performed optimally in cases of persistent spotting. This failure may be caused by the lack of a macroscopic landmark that indicates the correct resection area. This study has shown that there is a large variability concerning the extension and position of the uterine isthmus that makes it difficult to define a macroscopic landmark. In addition, our data suggests that the sacrouterine ligament is not a good landmark. In line with our findings, we refrain from defining surgical landmarks. Instead of landmarks we propose, in accordance with Schmidt et al. (13), a conical excision of the cervix but with the top of the conus positioned 10 mm away from the external orifice (Figure 4). In our opinion, this strategy better addresses the variance in isthmic area depth.

When conical deep cervical excision and minimising dissemination of sarcoma by precise diagnosis are practiced, we contend that LSH is still a very good method for removal of an uterus with benign disease.

## Conclusion

The extension of the physiological cervix uteri, as defined histologically by the presence of exclusively cervical glands, is often small. The isthmic region, containing both endometrial glands and cervical glands, shows a large range of variability, which may vary further in individual patients with the female cyclus. Remnant cervical endometrial glands appear to be associated with a low risk for later endometrial cancer, but with a higher risk of spotting. Conical excision of the cervix may go some way to solving the problem of the remaining cervical endometrial glands.

## Figures and Tables

**Table 1:**
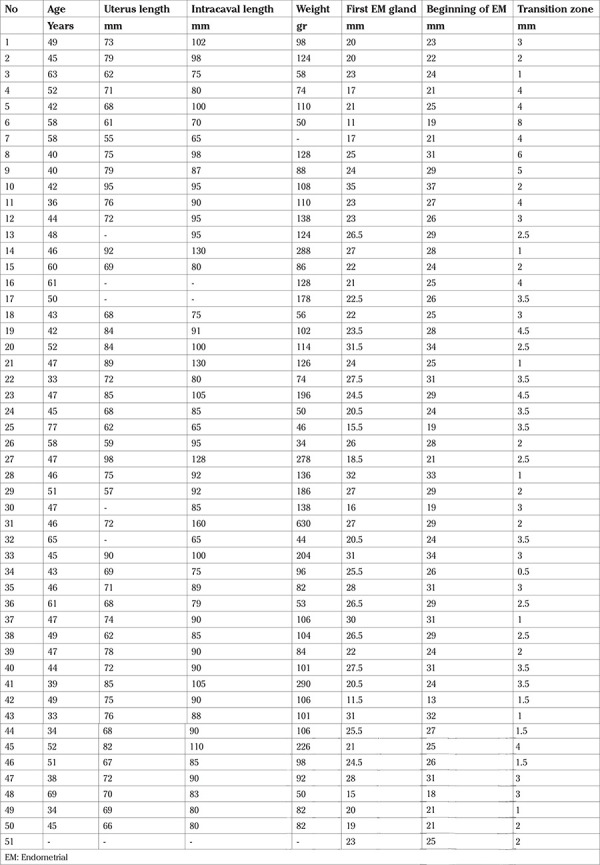
Patient data and histological details

**Figure 2a:**
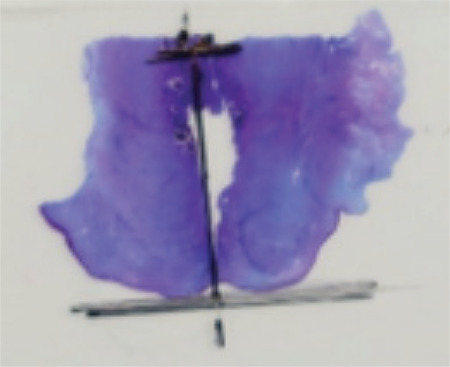
Longitudinal section of uterine cervix (one slide needed) A longitudinal section of uterine cervix is demonstrated. The first endometrial glands are present in this section. Thus, no second slide was needed. On the slide the cervical canal and reference line is marked for measurement

**Figure 2b:**
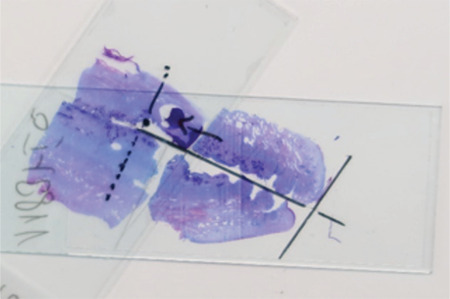
Longitudinal section of uterine cervix (two slides needed) In the first slide no endometrial glands were found. The second slide was positioned according to the line that marks cervical canal. The upper half was contiguous and could be used for measurement. The first endometrial cells were present more distally to the cervical canal than in Figure 2a

**Figure 3a:**
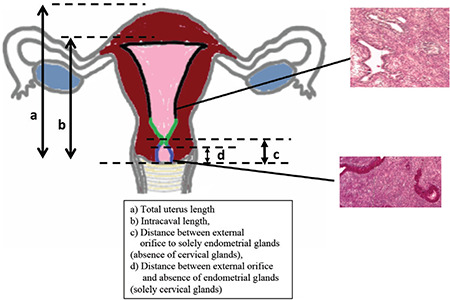
Reference lines Reference lines during the histological evaluation are demonstrated: (a) Total uterus length; (b) intracaval length; (c) distance between external orifice of the cervical channel and first exclusively endometrial gland area; (d) distance between external orifice of the cervical channel and area containing no endometrial glands

**Figure 3b:**
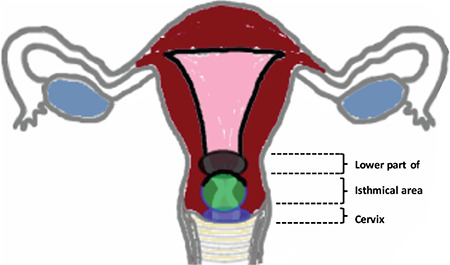
Clinical zones of uterus Schematic demonstration of the results of this study. Zones, rather than complex areas, were present in which specific cells were found

**Figure 3c:**
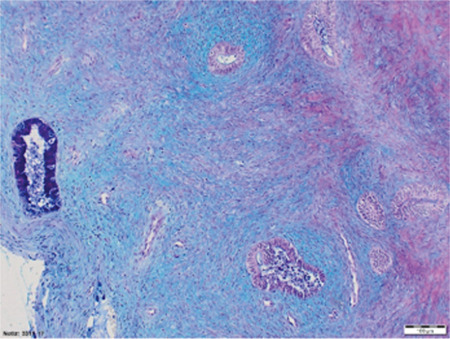
Isthmic zone Histological demonstration of the isthmus. Cervical glands (deep blue) and a few endometrial glands are coexistent
